# Prevalence of rare visceral aneurysms on 92,833 CAT scans (2005-2021)

**DOI:** 10.1590/1677-5449.202300742

**Published:** 2023-10-13

**Authors:** Sergio Quilici Belczak, Nathalia Almeida Cardoso da Silva, Matheus Toledo Nora, Paula Ribeiro do Prado Chadud, Luciana Helena Benetti, Camila de Freitas Corrêa, Adriano Tchibana, Ricardo Aun

**Affiliations:** 1 Instituto de Aprimoramento e Pesquisa em Angiorradiologia e Cirurgia Endovascular - IAPACE, São Paulo, SP, Brasil.; 2 Instituto Angiomar, São Luís, MA, Brasil.; 3 Hospital Israelita Albert Einstein, São Paulo, SP, Brasil.

**Keywords:** intra-abdominal aneurysms, visceral aneurysms, rare visceral aneurysms

## Abstract

**Background:**

Studies on the prevalence of rare visceral aneurysms are still scarce and the few studies that have focused on these aneurysms present prevalence rates in groups of patients with visceral aneurysms, but little is known about their prevalence in the general population.

**Objectives:**

To assess the prevalence of rare visceral aneurysms on CAT scans performed for diagnosis and follow-up of patients with other vascular pathologies.

**Methods:**

This cross-sectional study began by accessing all reports from CAT scans performed between January 2005 and July 2021 at a private hospital of excellence located in the city of São Paulo. A software program for pre-indexed reports was used to search the Radiological Information System (RIS) database to identify reports of patients with intra-abdominal aneurysms.

**Results:**

CAT scan reports from 92,883 patients were accessed. Of these, 2,597 (2.795%) showed intra-abdominal aneurysms, 937 (1.063%) of which were visceral, including 158 (0.171%) rare visceral aneurysms, which were more frequent among male patients and in the following segments: celiac trunk (0.098%), superior mesenteric (0.033%), left gastric (0.010%), pancreatic-duodenal (0.009%), and gastroduodenal arteries (0.005%) and the pancreatic arch (0.004%). Lower prevalence was found in other segments. Additional findings revealed concomitance of rare visceral aneurysms with other intra-abdominal aneurysms ranging from 11.11% to 66.67%.

**Conclusions:**

The prevalence of rare visceral aneurysms in a large population undergoing CAT scan was 0.171%, with greater involvement in male patients.

## INTRODUCTION

Although the prevalence of intra-abdominal aneurysms has received considerable research attention, there are oscillations between the results of different and, in particular with regard to rarer visceral aneurysms, and there are few studies with significant sample sizes.

Aneurysms of the celiac trunk appear to account for from 4.8 to 6.3% of all cases of visceral aneurysm,^1-4^ with a mild non-significant preponderance among men.^5^ In 40% of cases, they are concomitant with other visceral aneurysms. Graham et al.^5^ reported concomitance of celiac trunk aneurysms with aortic abdominal aneurysms (18%) and visceral aneurysms (38%).

While aneurysms of the superior mesenteric artery account for 3 to 5.5% of all visceral aneurysm cases,^6-8^ with no difference between the sexes and incidence predominantly among patients less than 50 years old,^9,10^ the prevalence of aneurysms of the inferior mesenteric artery is still unknown.^11^

Aneurysms of the visceral pancreatic-duodenal and gastroduodenal arteries and the gastric and gastroepiploic arteries are even rarer, with prevalence among men over 60 years of age. At the start of the 1970s, a study with 1,118 visceral aneurysm cases found an 8.0% incidence of aneurysms of the superior mesenteric artery and a 4.7% incidence for the gastric and gastroepiploic arteries.^12^ More recent studies have reported similar rates.^13-15^ However, the equivalent prevalence rates in the general population are unknown. In the majority of cases, these aneurysms involve the left gastric artery and aneurysms of the right artery are extremely rare.^16^

The objective of this study was to assess the prevalence of rarer visceral aneurysms on computed tomography angiographies ordered for diagnosis and follow-up of patients with different vascular pathologies.

## METHODS

This study was duly approved by the Research Ethics Committee at the Hospital Israelita Albert Einstein (consolidated opinion No. 5.081.688). This was a cross-sectional study that began by accessing all reports from computed tomography angiographies conducted from January 2005 to July 2021 at the Hospital Israelita Albert Einstein.

A dedicated software program was used to search pre-indexed reports in the hospital’s Radiological Information System (RIS) database to identify reports from patients with intra-abdominal aneurysms. Reports were excluded that referred to patients already included as were reports describing pseudoaneurysms. The data of interest from these reports (medical record number, date of angiotomography, patient sex and date of birth, and arteries with aneurysms) were input to a Microsoft Excel® spreadsheet. These data were analyzed descriptively. For the purposes of this study, rare visceral aneurysms were defined as those with prevalence rates less than 0.1% reported in the pertinent literature.

## RESULTS

**Figure 1:**
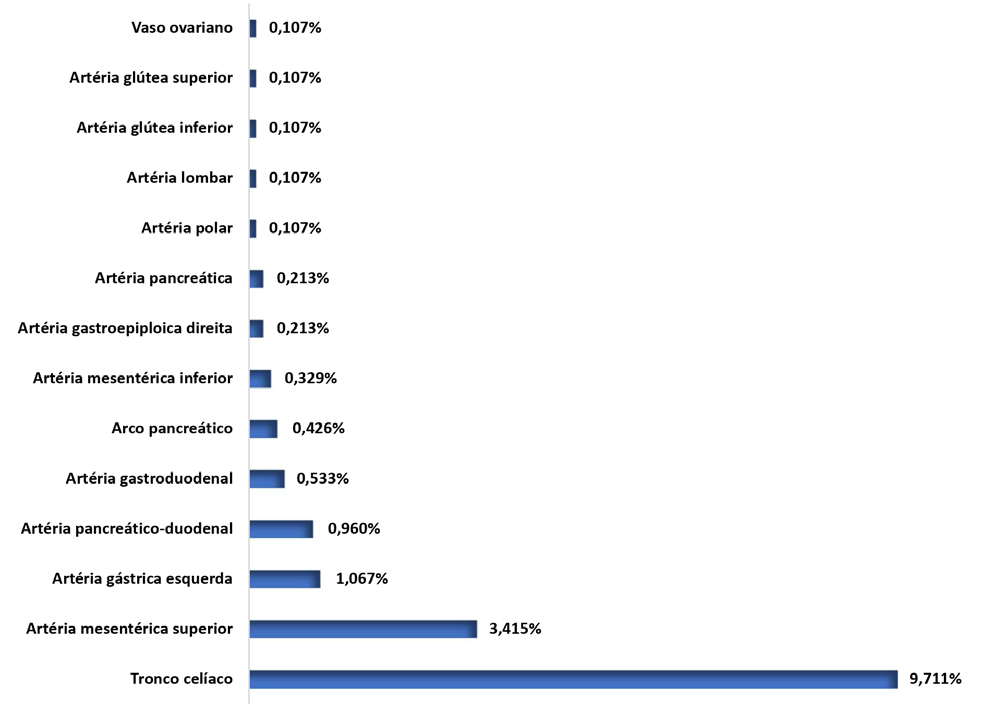
Distribution of frequencies of rare visceral aneurysms in 937 visceral aneurysms.

Angiotomography reports for 92,883 patients were accessed. Of these, 2,597 (2.795%) described intra-abdominal aneurysms, 937 (1.063%) of which were visceral, including 158 (0.171%) rare visceral aneurysms. These 158 patients had a total of 163 aneurysms, the majority of which were more prevalent among male patients (Table 1). The frequencies of these rare aneurysms among the total number of visceral aneurysms are illustrated in Figure 1, showing the prevalence of aneurysms of the celiac trunk followed by aneurysms of the superior mesenteric artery.

**Table 1:** Distribution of patients with 163 rare visceral aneurysms identified in 92,883 computed tomography angiography reports.

**Vessels involved by rare visceral aneurysms**	**Women**		**Men**		**Total**	
	**n**	**%**	**n**	**%**	**n**	**%**
Celiac trunk	8	0.008	83	0.090	91	0.098
Superior mesenteric artery	11	0.011	21	0.022	32	0.033
Left gastric artery	2	0.002	8	0.008	10	0.010
Pancreatic-duodenal artery	3	0.003	6	0.006	9	0.009
Gastroduodenal artery	1	0.001	4	0.004	5	0.005
Pancreatic arch	2	0.002	2	0.002	4	0.004
Inferior mesenteric artery	0	-	3	0.003	3	0.003
Right gastroepiploic artery	1	0.001	1	0.001	2	0.002
Pancreatic artery	0	-	2	0.002	2	0.002
Polar artery	1	0.001	0	-	1	0.001
Lumbar artery	1	0.001	0	-	1	0.001
Inferior gluteal artery	0	-	1	0.001	1	0.001
Superior gluteal artery	1	0.001	0	-	1	0.001
Ovarian vessel, right adnexal area	1	0.001	0	-	1	0.001
Totals	32	0.032	131	0.139	163	0.171

The mean age of patients with aneurysms of the celiac trunk (66.8±12.2) was similar (p = 0.532) among men (67.3±11.1) and women (61.6±19.7). The mean age of patients with aneurysms of the superior mesenteric artery (64.1±15.1) was also similar (p = 0.893) among men (63.7±13.8) and women (64.1±17.1). Mean ages of patients with less frequent rare aneurysms was 67.8±8.5 for cases with left gastric artery involvement; 57.6±13.3 for the pancreatic-duodenal artery; 69.8±13.9 for the gastroduodenal artery (one 87-year-old woman and four men aged from 50 to 84 years); and 58.3±12.8 for aneurysms of the pancreatic arch. The three men with aneurysms of the inferior mesenteric artery were aged 66, 73, and 81 years. Aneurysms of the right gastroepiploic artery were identified in one 66-year-old woman and one 73-year-old man. The two cases of aneurysmal pancreatic arteries were in men aged 52 and 56 years.

Multiple rare visceral aneurysms were identified in one case with three aneurysms of the pancreatic arch, one case with three aneurysms of the celiac trunk, one case with two aneurysms of the superior mesenteric artery, and one case with two aneurysms of the pancreatic-duodenal artery.

Concomitance of rare visceral aneurysms was observed in a 50-year-old man who had a gastroduodenal aneurysm and two aneurysms of the pancreatic-duodenal artery; in a 66-year-old man with an aneurysm of the celiac trunk and two aneurysms of the superior mesenteric artery; and in another 66-year-old man with three aneurysms of the celiac trunk, an aneurysm of the superior mesenteric artery, and an aneurysm of the gastroduodenal artery.

**Figure 2:**
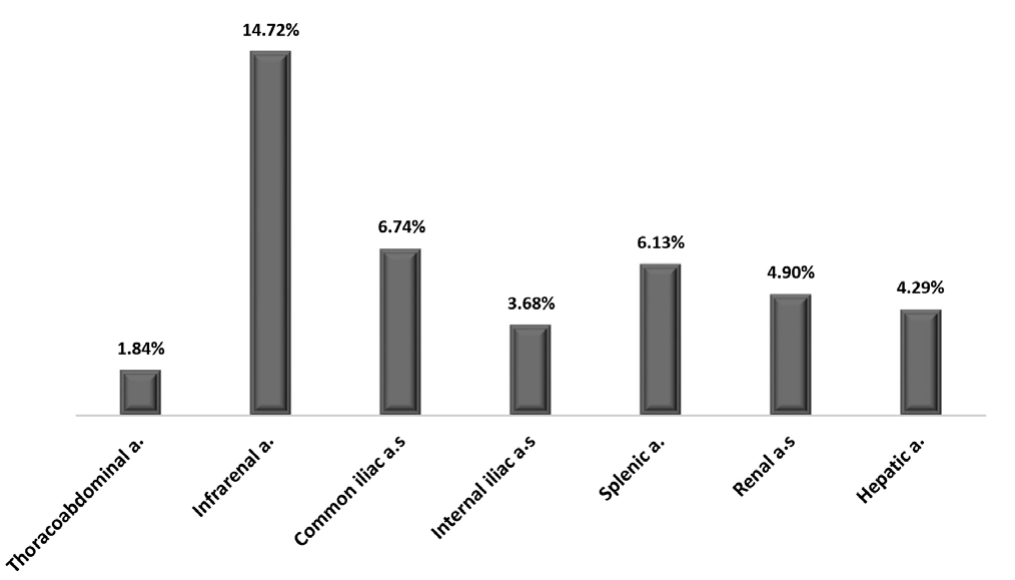
Distribution of frequencies of aortic, renal, and visceral artery aneurysms concomitant with rare visceral aneurysms (N = 163).

Forty-two (27.76%) of the 163 patients with rare visceral aneurysms also had concomitance with other intra-abdominal aneurysms, as detailed in Table 2. In general, the frequency of concomitance with other intra-abdominal aneurysms was greater among men, especially among those with aneurysms of the superior mesenteric artery. Exceptions were patients with aneurysms of the celiac trunk and of the gastroduodenal artery. The lowest frequency of concomitance (11.11%) was observed in patients with aneurysms of the pancreatic-duodenal artery and the highest frequency was in those with aneurysms of the left epigastric artery (66.67%). Table 3 lists aneurysmal intra-abdominal arteries with isolated or multiple concomitance with the most frequent rare visceral aneurysms, revealing a high frequency of concomitance of aneurysms of the left gastric artery with aneurysms of the infrarenal, common iliac, splenic, and hepatic arteries. Finally, Figure 2 illustrates the frequencies of aneurysmal intra-abdominal aortic, renal, and visceral (splenic and hepatic) arteries concomitant with the rarest visceral aneurysms identified in 163 patients.

**Table 2:** Distribution of patients with rare visceral aneurysms and other concomitant intra-abdominal aneurysms.

**Rare visceral aneurysms**	**Women**		**Men**		**Total**	
	**n/N**	**%**	**n/N**	**%**	**n/N**	**%**
Celiac trunk	2/8	25.00	16/83	19.27	18/91	19.78
Superior mesenteric a.	2/11	18.18	10/21	47.61	12/32	37.50
Left gastric a.	1/2	50.00	6/8	75.00	7/10	70.00
Pancreatic-duodenal a.	0/3	-	1/6	16.67	1/9	11.11
Gastroduodenal a.	1/1	100.00	0/4	-	1/5	20.00
Pancreatic arch	0/2	-	1/2	50.00	1/4	25.00
Mesenteric inferior a.	0/0	-	2/3	66.67	2/3	66.67

**Table 3:** Frequency distribution of percentages of patients by type of rare visceral aneurysm and concomitant intra-abdominal artery aneurysmal.

**Concomitant intra-abdominal aneurysmal arteries**	**Rare visceral aneurysms**						
	**CT**	**SMA**	**LGA**	**PDA**	**GDA**	**PA**	**IMA**
	**n = 91**	**n = 32**	**n = 10**	**n = 9**	**n = 5**	**n = 4**	**n = 3**
Thoracoabdominal a.	3.29%	-	-	-	-	-	-
Infrarenal a.	12.08%	15.62%	50.00%	11.11%	-	25.00%	33.33%
Common iliac a.s	5.49%	9.37%	30.00%	-	-	-	-
Internal iliac a.s	3.29%	3.13%	10.00%	-	-	-	33.33%
External iliac a.s	1.09%	-	-	-	-	-	-
Renal a.s	3.29%	12.50%	-	-	20.00%	-	-
Splenic a.	5.49%	18.75%	30.00%	11.11%	-	-	-
Hepatic a.	2.19%	3.13%	30.00%	11.11%	-	-	-

CT = celiac trunk; SMA = superior mesenteric artery; LGA = left gastric artery; PDA = pancreatic-duodenal artery; GDA = gastroduodenal artery; PA = pancreatic arch; IMA = inferior mesenteric artery.

No ruptured aneurysms were described in the angiotomography reports analyzed in this study.

## DISCUSSION

Our study enabled us to determine prevalence rates of rare visceral aneurysms in a significant number (92,833) of computed tomography angiographies performed over a period of 15.5 years. The few studies that have focused on these rare aneurysms present their prevalence rates among groups of patients with visceral aneurysms, but little was known about their prevalence in the general population. Our findings reveal a prevalence of 0.171% of rare visceral aneurysms, which is compatible with estimated prevalence rates of 0.01 to 0.20% reported in the literature.^17^

Aneurysms of the celiac trunk were identified in 0.098% of the computed tomography angiographies analyzed and in 9.711% of the visceral aneurysm cases, predominantly in male patients (91.20%). This prevalence within the subset of visceral aneurysms is superior to that reported in the literature (4.8 to 6.3%),^1-4^ while the high prevalence of men contradicts the information in the literature that there is no sex difference in the prevalence of this type of rare aneurysm.^5^ Rates of concomitance with other intra-abdominal aneurysms, both aortic (12.09%) and visceral (11.00%), were very much lower than the 18 and 38%, respectively, reported by Graham et al.^5^ in the mid-1980s. More recent studies do not report these data.

Aneurysms of the superior mesenteric artery were identified in 0.033% of all computed tomography angiographies analyzed and in 3.415% of the patients with visceral aneurysms. This last prevalence rate confirms the prevalence of 3 to 5.5% reported in the literature.^6-8^ Although the majority of patients with this type of rare aneurysm were male (65.62%), there was no significant difference between the sexes in relation to prevalence nor in relation to age, with mean age of 64.1±15.1 years. Our data once more confirm the results of other studies^9,10^ with regard to the proportional prevalence between men and women, but not in relation to age, since those studies reported higher prevalence in patients less than 50 years old. Just six of the 32 patients were under the age of 50 years, whereas 20 patients were aged from 60 to 88 years. We did not find data with which to compare our findings in relation to concomitance of aneurysms of the superior mesenteric artery with other aortic and visceral aneurysms. Aneurysms of the inferior mesenteric artery were identified in 0.003% of all computed tomography angiographies and in 0.329% of the patients with visceral aneurysms. These findings should help to improve knowledge of the prevalence of this type of aneurysm, which is still is considered unknown.^11^

Considering aneurysms of the gastric arteries (generally on the left, since aneurysms on the right are extremely rare and, indeed, no cases were identified in our study) and of the gastroepiploic artery, the reported prevalence is 4.7% of visceral aneurysms, but is unknown in the general population.^12-16^ Our findings revealed very much lower rates, since aneurysms of the left gastric artery (1.067%) and of the gastroepiploic artery (0.213%) accounted for 1.280% of the patients with visceral aneurysms. In the set of computed tomography angiographies analyzed, these prevalence rates were, respectively, 0.010 and 0.002%.

No data were found in the literature to support discussion of the remaining results of the present study, since practically all publications on other rare visceral aneurysms are case studies.

We believe that our findings should enrich the most up to date information on the prevalence rates of rare visceral aneurysms and their concomitance with other aortic, renal, and visceral aneurysms in the population undergoing computed tomography angiographies for other vascular diseases.
