# Supplementary material for: Prevalence of rare visceral aneurysms on 92,833 CAT scans (2005-2021)
Source: J Vasc Bras. 2023 Oct 13;22:e20230074. doi: 10.1590/1677-5449.202300742 (PMC10706156; doi:10.1590/1677-5449.202300742)
Supplement: Translation

# Translation: Prevalência de aneurismas viscerais raros em 92.833 angiotomografias (2005-2021)

**DOI:** 10.1590/1677-5449.202300741

**Authors:** Sergio Quilici Belczak, Nathalia Almeida Cardoso da Silva, Matheus Toledo Nora, Paula Ribeiro do Prado Chadud, Luciana Helena Benetti, Camila de Freitas Corrêa, Adriano Tchibana, Ricardo Aun

**Affiliations:** 1 Instituto de Aprimoramento e Pesquisa em Angiorradiologia e Cirurgia Endovascular - IAPACE, São Paulo, SP, Brasil.; 2 Instituto Angiomar, São Luís, MA, Brasil.; 3 Hospital Israelita Albert Einstein, São Paulo, SP, Brasil.

**Keywords:** aneurismas intra-abdominais, aneurismas viscerais, aneurismas viscerais raros

## Abstract

**Contexto:**

Ainda são incomuns os estudos de prevalência de aneurismas viscerais raros, e os poucos estudos que focalizaram esses aneurismas observam taxas de prevalência em grupos de pacientes com aneurismas viscerais, mas pouco se conhece sobre a sua prevalência na população geral.

**Objetivos:**

Avaliar a prevalência de aneurismas viscerais raros em angiotomografias realizadas para diagnóstico e seguimento de pacientes com diferentes patologias vasculares.

**Métodos:**

Este estudo transversal partiu do acesso a todos os laudos de angiotomografias realizadas entre janeiro de 2005 e julho de 2021 em hospital privado de excelência situado na cidade de São Paulo. Foi utilizado um programa de mecanismo de busca de laudos pré-indexados, cuja base de dados é o Sistema de Informação Radiológica (RIS), para acesso aos laudos de pacientes com aneurismas intra-abdominais.

**Resultados:**

Foram acessados laudos de angiotomografias de 92.883 pacientes, dos quais 2.597 (2,795%) apresentavam aneurismas intra-abdominais, sendo 937 (1,063%) viscerais, incluindo 158 (0,171%) aneurismas viscerais raros, mais frequente entre homens e nos seguintes segmentos: tronco celíaco (0,098%), artéria mesentérica superior (0,033%), artéria gástrica esquerda (0,010%), artéria pancreático-duodenal (0,009%), artéria gastroduodenal (0,005%) e arco pancreático (0,004%). Prevalências menores foram encontradas em outros diferentes segmentos. Achados adicionais revelaram taxa de concomitância de aneurismas viscerais raros com outros aneurismas intra-abdominais entre 11,11% e 66,67%.

**Conclusões:**

A prevalência de aneurismas viscerais raros em ampla população submetida a angiotomografias foi de 0,171%, com maior comprometimento nos pacientes do sexo masculino.

## INTRODUÇÃO

Embora a prevalência de aneurismas intra-abdominais venha sendo bastante estudada, há oscilações entre os estudos, e, especialmente no que tange aos aneurismas viscerais mais raros, os estudos com amostras significativas são escassos.

Os aneurismas do tronco celíaco parecem constituir entre 4,8 e 6,3% de todos os casos de aneurismas viscerais^1-4^ e com pequena preponderância não significativa entre os homens^5^ . Em 40% das vezes, são concomitantes a outros aneurismas viscerais. Graham et al.^5^ reportaram concomitância dos aneurismas de tronco celíaco com aneurismas abdominais aórticos (18%) e viscerais (38%).

Enquanto aneurismas da artéria mesentérica superior respondem por 3 a 5,5% de todos os casos de aneurismas viscerais^6-8^ , sem diferença entre os sexos e incidindo preferencialmente em pacientes com idade inferior a 50 anos^9-10^ , ainda é desconhecida a prevalência de aneurismas da artéria mesentérica inferior^11^ .

Ainda mais raros são os aneurismas dos ramos viscerais pancreático-duodenal e gastroduodenal e das artérias gástrica e gastroepiploica, prevalentes em homens com idade acima dos 60 anos. No início da década de 1970, em um estudo envolvendo 1.118 casos de aneurismas viscerais, foi encontrada incidência de 8,0% de aneurismas na artéria mesentérica superior e 4,7% nas artérias gástrica e gastroepiploica^12^ , e estudos mais recentes encontraram taxas semelhantes^13-15^ . Todavia, são desconhecidas essas prevalências na população geral. Na maioria dos casos, ocorrem aneurismas da artéria gástrica esquerda, sendo raríssimos os aneurismas da artéria direita^16^ .

Objetivou-se, neste estudo, avaliar a prevalência desses aneurismas viscerais mais raros em angiotomografias para diagnóstico e seguimento de pacientes com diferentes patologias vasculares.

## MÉTODOS

A realização deste estudo foi devidamente aprovada pelo Comitê de Ética em Pesquisa (CEP) do Hospital Israelita Albert Einstein (parecer consubstanciado n.º 5.081.688). Tratou-se de estudo transversal que partiu do acesso a todos os laudos de angiotomografias realizadas entre janeiro de 2005 e julho de 2021 no Hospital Israelita Albert Einstein.

Para tato, foi utilizado um programa de mecanismo de busca de laudos pré-indexados, cuja base de dados é o Sistema de Informação Radiológica (RIS), para acesso aos laudos de pacientes com aneurismas intra-abdominais. Foram excluídos laudos relativos a um mesmo prontuário/paciente, bem como laudos reportando presença de pseudoaneurismas. Os dados de interesse contidos nesses laudos (número do prontuário, data de realização da angiotomografia, data de nascimento e sexo do paciente e artérias com aneurisma) foram registrados em planilha do programa Microsoft Excel®. Esses dados foram analisados descritivamente. Para este estudo, foram considerados aneurismas viscerais raros aqueles com prevalências inferiores a 0,1% reportadas na literatura pertinente.

## RESULTADOS

Foram acessados laudos de angiotomografia de 92.883 pacientes, dos quais 2.597 (2,795%) apresentavam aneurismas intra-abdominais, sendo 937 (1,063%) viscerais, incluindo 158 (0,171%) aneurismas viscerais raros. Esses 158 pacientes apresentavam 163 aneurismas, a maioria deles mais prevalente no sexo masculino (Tabela 1). As frequências desses aneurismas raros entre os aneurismas viscerais estão representadas na Figura 1, na qual se observa prevalência dos aneurismas de tronco celíaco seguida dos aneurismas de artéria mesentérica superior.

**Tabela 1:** Distribuição de pacientes com 163 aneurismas viscerais raros identificados em 92.883 laudos de angiotomografias.

**Vasos acometidos por aneurismas viscerais raros**	**Mulheres**		**Homens**		**Total**	
	**n**	**%**	**n**	**%**	**n**	**%**
Tronco celíaco	8	0,008	83	0,090	91	0,098
Artéria mesentérica superior	11	0,011	21	0,022	32	0,033
Artéria gástrica esquerda	2	0,002	8	0,008	10	0,010
Artéria pancreático-duodenal	3	0,003	6	0,006	9	0,009
Artéria gastroduodenal	1	0,001	4	0,004	5	0,005
Arco pancreático	2	0,002	2	0,002	4	0,004
Artéria mesentérica inferior	0	-	3	0,003	3	0,003
Artéria gastroepiploica direita	1	0,001	1	0,001	2	0,002
Artéria pancreática	0	-	2	0,002	2	0,002
Artéria polar	1	0,001	0	-	1	0,001
Artéria lombar	1	0,001	0	-	1	0,001
Artéria glútea inferior	0	-	1	0,001	1	0,001
Artéria glútea superior	1	0,001	0	-	1	0,001
Vaso ovariano região anexial direita	1	0,001	0	-	1	0,001
Totais	32	0,032	131	0,139	163	0,171

**Figura 1:** Distribuição das frequências dos aneurismas viscerais raros em 937 aneurismas viscerais.

A média de idade dos pacientes com aneurismas no tronco celíaco (66,8±12,2) foi semelhante (p = 0,532) entre homens (67,3±11,1) e mulheres (61,6±19,7). Também a média de idade dos pacientes com aneurisma na artéria mesentérica superior (64,1±15,1) foi semelhante (p = 0,893) entre homens (63,7±13,8) e mulheres (64,1±17,1). As médias de idade dos pacientes com aneurismas raros menos frequentes foi de 67,8±8,5 nos casos de comprometimento da artéria gástrica esquerda; de 57,6±13,3 da artéria pancreático-duodenal; de 69,8±13,9 da artéria gastroduodenal (computando-se uma mulher de 87 anos e quatro homens com idades entre 50 e 84 anos); e de 58,3±12,8 em aneurismas do arco pancreático. Os três homens com aneurismas na artéria mesentérica inferior apresentavam idades de 66, 73 e 81 anos. Aneurismas na artéria gastroepiploica direita foram identificados em uma mulher com 66 anos e em um homem com 73 anos. Os dois casos de artéria pancreática aneurismática se referiram a homens com idades de 52 e 56 anos.

Foram identificados múltiplos aneurismas viscerais raros em um caso de três aneurismas no arco pancreático, um caso de três aneurismas no tronco celíaco, um caso de dois aneurismas na artéria mesentérica superior e um caso de dois aneurismas na artéria pancreático-duodenal.

Foi observada concomitância de aneurismas viscerais raros em um homem de 50 anos que apresentava um aneurisma gastroduodenal concomitante a dois aneurismas na artéria pancreático-duodenal; em um homem de 66 anos com um aneurisma do tronco celíaco concomitante a dois aneurismas na artéria mesentérica superior; e em um homem também com 66 anos com três aneurismas no tronco celíaco concomitantes a um aneurisma na artéria mesentérica superior mais um aneurisma na artéria gastroduodenal.

Dos 163 pacientes com aneurismas viscerais raros, 42 (27,76%) também apresentaram concomitância com outros aneurismas intra-abdominais, conforme demonstrado na Tabela 2. A frequência de concomitância com outros aneurismas intra-abdominais foi maior entre os homens, especialmente naqueles com aneurismas da artéria mesentérica superior, à exceção de pacientes com aneurismas em tronco celíaco e na artéria gastroduodenal. Menor frequência de concomitância (11,11%) foi observada em pacientes com aneurisma da artéria pancreático-duodenal, e maior frequência naqueles com aneurismas na artéria epigástrica esquerda (66,67%). Na Tabela 3, se encontram as artérias intra-abdominais aneurismáticas concomitantes isoladamente ou associadas aos aneurismas viscerais raros mais frequentes, revelando alta frequência de concomitância dos aneurismas da artéria gástrica esquerda com aneurismas das artérias infrarrenal, ilíacas comuns, esplênica e hepática. Por fim, na Figura 2, estão ilustradas as frequências de artérias intra-abdominais aórticas, renais e viscerais (esplênica e hepática) aneurismáticas concomitantes a aneurismas viscerais mais raros identificados em 163 pacientes.

**Tabela 2:** Distribuição de pacientes com aneurismas viscerais raros com outros aneurismas intra-abdominais concomitantes.

**Aneurismas viscerais raros**	**Mulheres**		**Homens**		**Total**	
	**n/N**	**%**	**n/N**	**%**	**n/N**	**%**
Tronco celíaco	2/8	25,00	16/83	19,27	18/91	19,78
A. mesentérica superior	2/11	18,18	10/21	47,61	12/32	37,50
A. gástrica esquerda	1/2	50,00	6/8	75,00	7/10	70,00
A. pancreático-duodenal	0/3	-	1/6	16,67	1/9	11,11
A. gastroduodenal	1/1	100,00	0/4	-	1/5	20,00
Arco pancreático	0/2	-	1/2	50,00	1/4	25,00
A. mesentérica inferior	0/0	-	2/3	66,67	2/3	66,67

**Tabela 3:** Distribuição das frequências percentuais de pacientes de acordo com o tipo de aneurisma visceral raro e a artéria intra-abdominal aneurismática concomitante.

**Artérias intra-abdominais aneurismáticas concomitantes**	**Aneurismas viscerais raros**						
	**TC**	**AMS**	**AGE**	**APD**	**AGD**	**AP**	**AMI**
	**n = 91**	**n = 32**	**n = 10**	**n = 9**	**n = 5**	**n = 4**	**n = 3**
A. toracoabdominal	3,29%	-	-	-	-	-	-
A. infrarrenal	12,08%	15,62%	50,00%	11,11%	-	25,00%	33,33%
A. ilíacas comuns	5,49%	9,37%	30,00%	-	-	-	-
A. ilíacas internas	3,29%	3,13%	10,00%	-	-	-	33,33%
A. ilíacas externas	1,09%	-	-	-	-	-	-
A. renais	3,29%	12,50%	-	-	20,00%	-	-
A. esplênica	5,49%	18,75%	30,00%	11,11%	-	-	-
A. hepática	2,19%	3,13%	30,00%	11,11%	-	-	-

TC = tronco celíaco; AMS = artéria mesentérica superior; AGE = artéria gástrica esquerda; APD = artéria pancreático-duodenal; AGD = artéria gastroduodenal; AP = arco pancreático; AMI = artéria mesentérica inferior.

**Figura 2:** Distribuição das frequências de artérias aórticas, renais e viscerais com aneurismas concomitantes a aneurismas viscerais raros (N = 163).

Não foi encontrado aneurisma roto nos laudos angiotomográficos incluídos neste estudo.

## DISCUSSÃO

Nosso estudo permitiu verificar as prevalências de aneurismas viscerais raros em um número importante (92.833) de angiotomografias realizadas ao longo de 15,5 anos. Os poucos estudos que focalizaram esses aneurismas raros trazem suas taxas de prevalência em grupos de pacientes com aneurismas viscerais, mas pouco se conhece sobre a prevalência na população geral. Assim, nossos achados revelaram prevalência de 0,171% de aneurismas viscerais raros, dado compatível com a prevalência estimada entre 0,01 e 0,20% reportada na literatura^17^ .

Aneurismas no tronco celíaco foram identificados em 0,098% das angiotomografias analisadas e em 9,711% dos aneurismas viscerais, predominantemente em pacientes do sexo masculino (91,20%). Essa prevalência no grupo de aneurismas viscerais é superior à relatada na literatura (4,8 a 6,3%)^1-4^ , e a grande prevalência de homens vai de encontro à informação da literatura de que não há diferença na prevalência desse tipo de aneurisma raro entre os sexos^5^ . As taxas de concomitância com outros aneurismas intra-abdominais tanto aórticos (12,09%) quanto viscerais (11,00%) foram bastante inferiores aos 18 e 38%, respectivamente, relatados por Graham et al.^5^ em meados da década de 1980. Estudos mais recentes não trazem essa informação.

Aneurismas da artéria mesentérica superior foram identificados em 0,033% de todas as angiotomografias analisadas e em 3,415% dos pacientes com aneurismas viscerais; essa última prevalência ratifica a prevalência de 3 a 5,5% relatada na literatura^6-8^ . Embora a maioria dos pacientes com esse tipo de aneurisma raro fosse do sexo masculino (65,62%), não houve diferença significativa entre os sexos em relação à prevalência nem em relação à idade, cuja média foi de 64,1±15,1 anos. Nossos dados, novamente, ratificam resultados de outros estudos^9-10^ no que tange à prevalência proporcional entre homens e mulheres, mas não em relação à idade, já que esses estudos apontam prevalência em pacientes com idade inferior a 50 anos. Apenas seis dos 32 pacientes apresentaram idade inferior a 50 anos, enquanto 20 pacientes tinham idades entre 60 e 88 anos. Não encontramos dados para discutir nossos achados em relação à concomitância dos aneurismas na artéria mesentérica superior com outros aneurismas aórticos e viscerais. Aneurismas na artéria mesentérica inferior foram identificados em 0,003% de todas as angiotomografias e em 0,329% dos pacientes com aneurismas viscerais. Esses achados podem auxiliar a conhecer melhor a prevalência desse tipo de aneurisma, já que ainda é considerada desconhecida^11^ .

Considerando-se aneurismas nas artérias gástrica (geralmente a esquerda, já que aneurismas na direita são extremamente raros e, de fato, não foi identificado nenhum caso em nosso estudo) e gastroepiploica, a prevalência relatada é de 4,7% dos aneurismas viscerais, mas desconhecida na população geral^12-16^ . Nossos achados revelaram taxas bastantes inferiores, já que aneurismas da artéria gástrica esquerda (1,067%) e aneurismas da artéria gastroepiploica (0,213%) responderam por 1,280% dos pacientes com aneurismas viscerais. No conjunto de angiotomografias, essas prevalências foram de, respectivamente, 0,010 e 0,002%.

Não foram encontrados dados para sustentar a discussão dos demais resultados deste estudo, visto que praticamente a totalidade de publicações sobre os demais aneurismas viscerais raros é formada por estudos de caso.

Acreditamos que nossos achados possam enriquecer o conhecimento mais atual das prevalências dos aneurismas viscerais raros, bem como de sua concomitância com outros aneurismas aórticos, renais e viscerais em população submetida a angiotomografias por diferentes doenças vasculares.
